# Optimal Coverage of Full Frequency Reuse in FFR Networks in Relation to Power Scaling of a Base Station

**DOI:** 10.3390/s23218925

**Published:** 2023-11-02

**Authors:** Minyoung Seo, Seok-Ho Chang, Jong-Man Lee, Ki-Hun Kim, Hyun Park, Sang-Hyo Kim

**Affiliations:** 1 Department of Smart ICT Convergence, Konkuk University, Seoul 05029, Republic of Korea; ing06060@konkuk.ac.kr (M.S.); seokho@konkuk.ac.kr (S.-H.C.); 2 Hanwha Systems, Sungnam 13524, Republic of Korea; jongman0319.lee@hanwha.com (J.-M.L.); kihun19.kim@hanwha.com (K.-H.K.); h82.park@hanwha.com (H.P.); 3 College of Information and Communication Engineering, Sungkyunkwan University, Suwon 16419, Republic of Korea

**Keywords:** base station power, cellular networks, fractional frequency reuse (FFR), full FR coverage, multiple-input multiple-output (MIMO), orthogonal space–time block codes (OSTBC), outage probability, vertical Bell Labs space–time architecture (V-BLAST)

## Abstract

As a strategy to coordinate inter-cell interference in cellular networks, a fractional frequency reuse (FFR) system is proposed, in which the frequency bandwidth is split into two orthogonal bands; users staying near the center of a FFR cell use the band with a frequency reuse (FR) factor of one (i.e., full FR), and users located close to the cell edge utilize the band with a FR factor greater than one (i.e., partial FR). Full FR coverage, which identifies full FR and partial FR regions (that is, near-center and near-edge regions) within a FFR cell, has a crucial effect on system performance. Some of the authors of this paper recently investigated the optimization of full FR coverage to maximize system throughput. They analytically showed that under the constraint of satisfying a specified target outage probability, the optimal full FR coverage is a non-increasing function of base station power when all base station powers in the cellular network are scaled at an equal rate. Interestingly, in this paper, it is proven that as the power of a single base station is scaled, the optimal full FR coverage in that cell is a non-decreasing function of base station power. Our results provide useful insight into the design of full FR coverage in relation to the transmit power of a base station. It gives a deeper understanding of the intricate relationship between important FFR system parameters of base station power and full FR coverage.

## 1. Introduction

**Figure 1:**
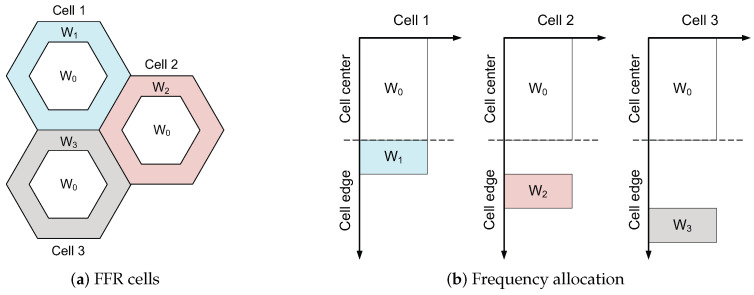
An example of FFR systems with the partial FR factor of M=3. (**a**) $W_{0}$ indicates the frequency band for the full FR region (near-center region); $W_{1}$, $W_{2}$, and $W_{3}$ denote the bands for the partial FR region (near-edge region) for Cells 1, 2, and 3, respectively. (**b**) For each cell, the x-axis indicates the power spectral density, and the y-axis indicates frequency.

To achieve large system throughput, base stations are densely deployed in recent cellular networks. When users within a cell have resources orthogonal to each other, the primary source of interference is inter-cell interference. Inter-cell interference coordination [1,2] is a method used to improve system performance in such cellular systems, and a fractional frequency reuse (FFR) strategy is suggested as a simple inter-cell interference coordination scheme for orthogonal frequency-division multiplexing access (OFDMA)-based cellular networks [3]. We let *M* denote the frequency reuse (FR) factor. In the FFR system, the frequency bandwidth is split into two orthogonal bands so that users located close to the center of a cell utilize the band with M=1, which is referred to as full FR, and those near the edge of a cell use the band with M>1, which yields partial FR. As a result, a FFR cell is split into full FR and partial FR regions. An example of the FFR systems is shown in Figure 1. Full FR coverage, also called the distance threshold, serves to differentiate between full FR and partial FR regions within a cell. Full FR coverage has a significant effect on FFR networks performance [3,4,5,6,7,8,9,10,11,12,13,14,15].

In this paper, we study the optimal full FR coverage that maximizes system throughput in multiple-input multiple-output (MIMO) cellular networks. Studies on the optimal full FR coverage were conducted by many researchers [3,4,5,6,7,8,9,10,11]. For instance, in [3], the presence of a correlation between subbands in OFDM is considered, and system throughput is evaluated as a function of full FR coverage; it is shown that when the full FR coverage increases to a certain point, there is a corresponding increase in system throughput. In [7], the authors investigated the optimal full FR coverage through numerical evaluation in order to maximize the area spectral efficiency. The work in [9] studied a framework for assessing system throughput and cell coverage in the uplink of FFR cellular networks. It was shown that by optimally selecting full FR coverage and FR factor in relation to power control parameters, both cell coverage and system throughput can be enhanced. In [10], an optimal full FR coverage was determined by observing the number of users and the number of subcarriers in OFDMA systems. In addition to the aforementioned works [3,4,5,6,7,8,9,10,11], previous studies such as [12,13,14,15] showed the crucial role of full FR coverage in the design of FFR systems. These studies demonstrated that system performance, such as sum capacity and coverage probability, is significantly affected by the selection of full FR coverage.

In general, base station power and cell coverage are closely related system parameters [16,17,18,19,20]. In the literature, including [3,4,5,6,7,8,9,10,11,12,13,14,15,16,17,18,19,20], however, the impact of base station power on the optimal full FR coverage has not been studied analytically. For instance, in [7,8], such an impact was observed only from the simulation or numerical evaluation of performance metrics. In [21], the optimal full FR coverage was mathematically analyzed by some of the authors of this paper, but it was investigated in terms of cell size rather than base station power for size-scalable aerial communication networks. Recently, in [22,23], some of the authors of this paper analyzed the impact of base station power on the optimal full FR coverage for maximizing system throughput, with the constraint of meeting a specified target outage probability. It was proven in [22,23] that the optimal full FR coverage is a *non-increasing function of base station power* when the power of all base stations in the network is scaled up or down at an equal rate. When optimizing cellular networks, however, it is more common to fine-tune the power of individual base stations rather than adjusting the power of all base stations. In this paper, we address such issues and prove that when the power of a single base station is scaled up or down, the optimal full FR coverage in that cell is *a non-decreasing function of the base station power*. This is interesting in the sense that these results are the opposite of those in [22,23].

The MIMO technique is a significant advancement in wireless communications, offering improvements in link reliability and data rates. Spatial diversity techniques, such as orthogonal space–time block codes (OSTBCs), harness diversity gains from the communication channels to enhance reliability. On the other hand, spatial multiplexing schemes, exemplified by the vertical Bell Laboratories layered space–time architecture (V-BLAST), employ a layered approach to enhance data rates [24]. The results in this paper were derived for both MIMO systems of OSTBC and V-BLAST with a zero-forcing linear receiver, while only OSTBC was considered in [22,23] for the mathematical analysis. Our results provide useful system design guidelines when initially planning a cellular FFR network in terms of base station power and full FR coverage. In addition, when the base station power changes in self-organizing networks [25] or aerial communication networks [26], or when the base station power is optimized in terms of energy consumption in green wireless networks [27], our analysis gives useful insight into the design of the full FR coverage in relation to power. The rest of this paper is organized as follows. In Section 2, some technical preliminaries are provided. In Section 3, we analyze the optimal full FR coverage in relation to power of a base station. In Section 4, we offer numerical results and discussions, and in Section 5 we conclude our work.

## 2. Preliminaries

### 2.1. Cellular System Model

**Figure 2:**
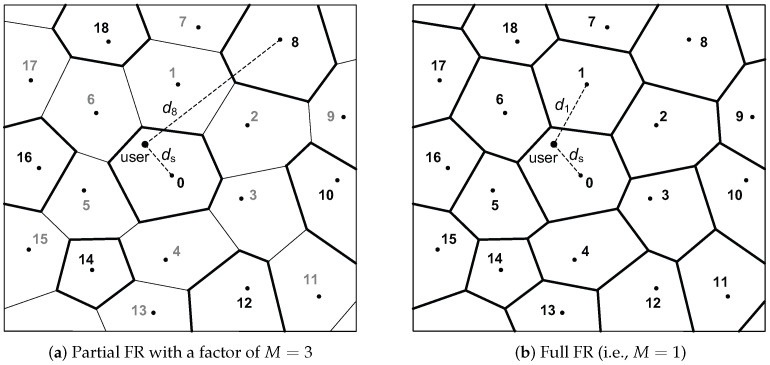
An example of cellular networks with an irregular layout of base stations. The serving cell is identified as cell 0, and cells with bold boundaries are indicative of interfering cells. (**a**) When M=3, cells labeled 8, 10, 12, 14, 16, and 18 are designated as interfering cells. (**b**) In the case of M=1, all neighboring cells (i.e., cells labeled 1, 2, …, 18) are classified as interfering cells.

This paper considers downlink transmission in a MIMO cellular network of diverse topologies, encompassing hexagonal and linear cellular models, while accommodating arbitrary base station deployments. We make the assumption that in both scenarios, whether it is full or partial FR, users within a given cell utilize orthogonal resources and do not cause interference to each other. A *serving cell* refers to the cell engaged in communication with a user, while all cells other than the serving cell are denoted as *neighboring cells*. An *interfering cell* is specifically a neighboring cell that operates on the same frequency band as the serving cell. An illustration of such cellular networks is provided in Figure 2. We let $d_{i}$ denote the distance between the base station of the *i*th neighboring cell and the user within the serving cell. Additionally, we use $p_{i}$ to represent the transmit power of the base station in the *i*th neighboring cell. Subsequently, the power of the signal received by the user can be expressed as $p_{i}k_{0}{\left(d_{\mathrm{ref}}/d_{i}\right)}^{\alpha }\beta_{i}$, where $k_{0}$ is a dimensionless constant, $d_{\mathrm{ref}}$ represents a reference distance for the antenna’s far field, α denotes the path-loss exponent, and $0<\beta_{i}<1$ accounts for attenuation due to shadow fading.

We can express the power of inter-cell interference as [22]

(1) $$P_{\mathrm{ici}}=\sum_{i=1}^{N_{\mathrm{cell}}}p_{i}k_{0}{\left(\frac{d_{\mathrm{ref}}}{d_{i}}\right)}^{\alpha }\beta_{i}I_{M}\left(i\right),$$

where $N_{\mathrm{cell}}$ is the number of neighboring cells (that is, all cells in the network except the serving cell), and $I_{M}\left(i\right)$ is an indicator function defined as follows: $I_{M}\left(i\right)=1$ for $i\in S_{M}$, where the set of $S_{M}$ comprises all interfering cells with the FR factor of *M*; $I_{M}\left(i\right)=0$ otherwise. Note that $S_{M}$ includes not only the closest interfering cells but encompasses all interfering cells in the entire network. In the case of full FR, all neighboring cells are considered interfering cells, leading to $I_{1}\left(i\right)=1$ ($i=1,\ldots ,N_{\mathrm{cell}}$).

### 2.2. Channel Model

For the downlink transmission, we consider a system model with $N_{\mathrm{tx}}\left(\geq 1\right)$ transmit and $N_{\mathrm{rx}}\left(\geq 1\right)$ receive antennas. Let $N_{\mathrm{uc}}$ represent the number of uncoded complex symbols, denoted as $\left\{u_{1},\ldots ,u_{N_{\mathrm{uc}}}\right\}$, that are mapped to a space–time codeword, $S=[s\left(1\right)\cdots s\left(T_{s}\right)]$, where S is of size $N_{\mathrm{tx}}\times T_{s}$. This codeword is transmitted over $T_{s}$ symbol durations through $N_{\mathrm{tx}}$ transmit antennas. We designate $p_{s}$ as the transmit power of the base station in the serving cell, and $d_{s}$ as the distance between the user and the base station within the serving cell. Additionally, we denote the relevant shadowing attenuation as $\beta_{s}$. The baseband equivalent model at the *k*th time symbol duration ($k=1,\ldots ,T_{s}$) is then expressed as [22]

(2) $$\;y\left(k\right)=\sqrt{k_{0}{\left(\frac{d_{\mathrm{ref}}}{d_{s}}\right)}^{\alpha }\beta_{s}}\;Hs\left(k\right)+z\left(k\right)+n\left(k\right),$$

where y(k) represents an $N_{\mathrm{rx}}\times 1$ received signal vector, s(k) is an $N_{\mathrm{tx}}\times 1$ transmitted signal vector, z(k) is an $N_{\mathrm{rx}}\times 1$ inter-cell interference signal vector, and n(k) is an $N_{\mathrm{rx}}\times 1$ noise vector. The channel matrix H is $N_{\mathrm{rx}}\times N_{\mathrm{tx}}$, where the entry $h_{ij}$ corresponds to the complex channel gain between the *j*th transmit antenna and the *i*th receive antenna; all entries are assumed to be independent and identically distributed (i.i.d.) following a complex normal distribution with zero mean and unit variance ∼CN(0,1). We assume that the matrix H is known to the receiver but not to the transmitter; that is, that channel state information is solely accessible at the receiver. Further, it is assumed that H is random but remains constant over a duration of $T_{s}$ symbol period, which implies a quasi-static Rayleigh i.i.d. fading model.

In (2), the transmitted signal vector satisfies $E\left[s(k)\right]=0_{N_{\mathrm{tx}}\times 1}$ and $\frac{1}{T_{s}}\sum_{k=1}^{T_{s}}E\left[{∥s\left(k\right)∥}^{2}\right]$$=p_{s}$, where E[·] denotes expectation, and $0_{m\times n}$ is the zero matrix of size m×n. Let ${\left(\cdot \right)}_{i}$ denote the *i*th component of a vector. For non-zero encoded complex symbols, i.e., ${\left(s\left(k\right)\right)}_{i}\neq 0$, it follows that $E\left[{|\left(s\left(k\right)\right)}_{i}{|}^{2}\right]=p_{s}T_{s}/N_{c}$ ($1\leq i\leq N_{\mathrm{tx}}$, $1\leq k\leq T_{s}$), where $N_{c}$ represents the number of non-zero encoded symbols within a space–time codeword $S=[s\left(1\right)\cdots s\left(T_{s}\right)]$. The spatial multiplexing rate, denoted as $r_{s}$, is defined as the ratio of the number of uncoded symbols $\left\{u_{1},\ldots ,u_{N_{\mathrm{uc}}}\right\}$ contained within a space–time codeword to its time duration $T_{s}$. That is, $r_{s}$ is expressed as $r_{s}≜N_{\mathrm{uc}}/T_{s}$. In the case of OSTBC, $r_{s}$ is given by the expression $r_{s}=N_{c}/\left(N_{\mathrm{tx}}T_{s}\right)$ [28]. For $N_{\mathrm{tx}}=2$, the Alamouti scheme achieves a spatial multiplexing rate of $r_{s}=1$.

Regarding the inter-cell interference signal vector z(k), it is represented by [22]

(3) $$z\left(k\right)=\sum_{i=1}^{N_{\mathrm{cell}}}\sqrt{k_{0}{\left(\frac{d_{\mathrm{ref}}}{d_{i}}\right)}^{\alpha }\beta_{i}}\;I_{M}\left(i\right)\;H_{i}s_{i}\left(k\right),$$

where $H_{i}$ is the $N_{\mathrm{rx}}\times N_{\mathrm{tx}}$ channel matrix, which represents the complex channel gain between the base station in the *i*th neighboring cell and the user; the description of H, mentioned below (2), also applies to $H_{i}$. $s_{i}\left(k\right)$ is the $N_{\mathrm{tx}}\times 1$ signal vector transmitted from the base station in the *i*th neighboring cell. It satisfies $E\left[s_{i}\left(k\right)\right]=0_{N_{\mathrm{tx}}\times 1}$ and $\frac{1}{T_{s}}\sum_{k=1}^{T_{s}}E\left[∥s_{i}{\left(k\right)∥}^{2}\right]=p_{i}$ (recall that $p_{i}$ represents the transmit power of the base station in the *i*th neighboring cell). From (3), we have $E\left[z(k)\right]=0_{N_{\mathrm{rx}}\times 1}$, which is attained since $H_{i}$ and $s_{i}\left(k\right)$ are statistically independent. Additionally, we obtain $E\left[{∥z\left(k\right)∥}^{2}\right]=\sum_{i=1}^{N_{\mathrm{cell}}}k_{0}{\left(d_{\mathrm{ref}}/d_{i}\right)}^{\alpha }\beta_{i}\;I_{M}\left(i\right)\;E\left[{\left(s_{i}\left(k\right)\right)}^{H}H_{i}^{H}H_{i}s_{i}\left(k\right)\right]=N_{\mathrm{rx}}\sum_{i=1}^{N_{\mathrm{cell}}}k_{0}{\left(d_{\mathrm{ref}}/d_{i}\right)}^{\alpha }\beta_{i}\;I_{M}\left(i\right)\;E\left[∥s_{i}{\left(k\right)∥}^{2}\right]$, where the first equality results from the independence of $H_{i}s_{i}\left(k\right)$ from $H_{j}s_{j}\left(k\right)$ for i≠j, and the second equality follows from the property $E\left[H_{i}^{H}H_{i}\right]=N_{\mathrm{rx}}I_{N_{\mathrm{tx}}}$, where ${\left(\cdot \right)}^{H}$ denotes the Hermitian operation, and $I_{m}$ represents the m×m identity matrix. Therefore, we obtain $\frac{1}{T_{s}}\sum_{k=1}^{T_{s}}E\left[{∥z\left(k\right)∥}^{2}\right]/N_{\mathrm{rx}}=\sum_{i=1}^{N_{\mathrm{cell}}}p_{i}k_{0}{\left(d_{\mathrm{ref}}/d_{i}\right)}^{\alpha }\beta_{i}\;I_{M}\left(i\right)$. This expression provides the power of the inter-cell interference, denoted as $P_{\mathrm{ici}}$ as given in (1). Note that $N_{\mathrm{cell}}$ is typically a large number, and the number of transmit antennas in the base station is often greater than one. Therefore, based on these factors and the central limit theorem, we make the assumption that the inter-cell interference signal follows a Gaussian distribution.

The noise vector n(k)$∼\mathrm{CN}(0,\sigma_{n}^{2}I_{N_{\mathrm{rx}}})$ is characterized as a zero-mean, circularly symmetric complex Gaussian noise with the property $E\left[n\left(k\right)n{\left(l\right)}^{H}\right]$=$\sigma_{n}^{2}I_{N_{\mathrm{rx}}}\delta \left(k-l\right)$$\left(1\leq k,l\leq T_{s}\right)$, where δ(·) denotes the Kronecker delta function. The single-sided power spectral density of the elements of n(k) is denoted as $N_{0}$. Consequently, we have $\sigma_{n}^{2}=N_{0}B/M$, where *B* represents the frequency bandwidth allocated per user within a cell employing the FR factor of *M*, and B/M signifies the bandwidth available for each user to utilize.

## 3. Analysis of Full FR Coverage in Relation to Power Scaling of a Base Station

First, we consider the case where multiple-input single-output (MISO) or MIMO systems of OSTBC (i.e., $N_{\mathrm{tx}}\geq 2$, $N_{\mathrm{rx}}\geq 1$) are employed for the downlink transmission in the serving cell.

### 3.1. MISO or MIMO Systems of OSTBC

To decode a set of symbols, $\left\{u_{1},\ldots ,u_{N_{\mathrm{uc}}}\right\}$, encoded by OSTBC, the space–time matched filter is employed [28] (Section 5.5.4), [29] (Section 7.4). The MIMO channel H is then transformed into $N_{\mathrm{uc}}$ equivalent single-input single-output (SISO) channels with gain ${∥H∥}_{F}^{2}$, where ${∥\cdot ∥}_{F}$ denotes the Frobenius norm. From this and (2), the sufficient statistic of $u_{i}$, denoted by ${\hat{u}}_{i}$, is given by [28] (Section 5.5.4), [29] (Section 7.4).

(4) $${\hat{u}}_{i}=\sqrt{k_{0}{\left(\frac{d_{\mathrm{ref}}}{d_{s}}\right)}^{\alpha }\beta_{s}}\;{∥H∥}_{F}^{2}\;u_{i}+z_{i}+n_{i},\;i=1,\ldots ,N_{\mathrm{uc}},$$

where $z_{i}∼{\mathrm{CN}(0,∥H∥}_{F}^{2}P_{\mathrm{ici}})$ is the inter-cell interference (recall that $P_{\mathrm{ici}}$, given by (1), is the power of the inter-cell interference, and refer to Section 2.2 for Gaussian assumption) and $n_{i}∼{\mathrm{CN}(0,∥H∥}_{F}^{2}N_{0}B/M)$ is the noise. For a set of uncoded symbols, $\left\{u_{1},\ldots ,u_{N_{\mathrm{uc}}}\right\}$, we have $E\left[u_{i}\right]=0$ and $E\left[|u_{i}{|}^{2}\right]=p_{s}/\left(r_{s}N_{\mathrm{tx}}\right)$, where the second equality follows from the fact that $E\left[|u_{i}{|}^{2}\right]=E\left[{|\left(s\left(k\right)\right)}_{j}{|}^{2}\right]=p_{s}T_{s}/N_{c}$ ($1\leq j\leq N_{\mathrm{tx}}$, $1\leq k\leq T_{s}$) for a non-zero encoded symbol ${\left(s\left(k\right)\right)}_{j}\neq 0$, and that $r_{s}=N_{c}/\left(N_{\mathrm{tx}}T_{s}\right)$ for OSTBC (refer to Section 2.2). From this, (1), and (4), for MISO/MIMO systems of OSTBC, the post-processing signal-to-interference and noise ratio (SINR) at the user can be expressed as [28] (Equation (5.162)).

(5) $$\mathrm{SINR}=\frac{\;\;\frac{1}{r_{s}N_{\mathrm{tx}}}p_{s}k_{0}{\left(\frac{d_{\mathrm{ref}}}{d_{s}}\right)}^{\alpha }\beta_{s}{∥H∥}_{F}^{2}\;\;}{\frac{N_{0}B}{M}+\sum_{i=1}^{N_{\mathrm{cell}}}p_{i}k_{0}{\left(\frac{d_{\mathrm{ref}}}{d_{i}}\right)}^{\alpha }\beta_{i}I_{M}\left(i\right)}\;.$$

In the following, the outage probabilities for both full FR and partial FR are derived, and they are compared under the condition that their data rates are identical. Note that, with appropriately powerful channel codes, the error probability when not in an outage situation is very low. Consequently, the outage probability serves as an accurate approximation of the actual packet error probability [30] With this, we analyze the effect of the transmit power of a base station on the optimal full FR coverage. In this analysis, we make the assumption that the user’s location within a cell remains fixed; more precisely, changes occur only on the order of the signal wavelength. As a result, *large-scale propagation effects, such as path loss and shadow fading, are considered constant, while the small-scale multipath fading channel varies randomly.* With this configuration, we examine whether a user’s location within a cell shifts between the partial FR region and the full FR region, or vice versa, as the power of the base station changes. Using (5), the outage probability for OSTBC can be written as a function of $p_{s}$ as [22]

(6) $$P_{\mathrm{ostbc}}\left(p_{s}\right)=\mathrm{Pr}\left[\frac{r_{s}B}{M}\log_{2}\left(1+\frac{\frac{1}{r_{s}N_{\mathrm{tx}}}p_{s}k_{0}{\left(\frac{d_{\mathrm{ref}}}{d_{s}}\right)}^{\alpha }\beta_{s}{∥H∥}_{F}^{2}}{\frac{N_{0}B}{M}+\sum_{i=1}^{N_{\mathrm{cell}}}p_{i}k_{0}{\left(\frac{d_{\mathrm{ref}}}{d_{i}}\right)}^{\alpha }\beta_{i}I_{M}\left(i\right)}\right)<R\right],$$

where *R* represents the data rate (bits/s) allocated to the downlink transmission, and the channel capacity formula is applied under the assumption that the components of each codeword in the transmitted signal are i.i.d. and follow a Gaussian distribution. We recall that the channel matrix H is random, but the path loss and shadow fading remain constant, meaning that $d_{s}$, $\beta_{s}$, $d_{i}$, and $\beta_{i}$ are unchanging. Equation (6) can be rewritten as [22]

(7) $$P_{\mathrm{ostbc}}\left(p_{s}\right)=\mathrm{Pr}\left[{∥H∥}_{F}^{2}<\frac{r_{s}N_{\mathrm{tx}}d_{s}^{\alpha }}{p_{s}\beta_{s}}\left(\frac{N_{0}B}{Mk_{0}d_{\mathrm{ref}}^{\alpha }}+\sum_{i=1}^{N_{\mathrm{cell}}}\frac{p_{i}\beta_{i}}{d_{i}^{\alpha }}\;I_{M}\left(i\right)\right)\cdot \left(2^{\frac{MR}{r_{s}B}}-1\right)\right].$$

The cumulative distribution function (CDF) of ${∥H∥}_{F}^{2}$, characterized as a chi-square random variable with $2N_{\mathrm{tx}}N_{\mathrm{rx}}$ degrees of freedom, is expressed as $F_{{∥H∥}_{F}^{2}}\left(x\right)=1-e^{-x}\left(\sum_{n=1}^{N_{\mathrm{tx}}N_{\mathrm{rx}}}x^{n-1}/\left(n-1\right)!\right)$. Let $P_{\mathrm{ostbc},\;f}\left(p_{s}\right)$ and $P_{\mathrm{ostbc},\;p}\left(p_{s}\right)$ represent the outage probabilities for full FR and partial FR, respectively. If we set *M* to 1 for full FR and m(>1) for partial FR, combining (7) with the CDF of ${∥H∥}_{F}^{2}$ results in [22]

$$\begin{array}{ccc} & & \;P_{\mathrm{ostbc},\;f}\left(p_{s}\right)=1-\exp\left(-\frac{r_{s}N_{\mathrm{tx}}d_{s}^{\alpha }}{p_{s}\beta_{s}}\left(\frac{N_{0}B}{k_{0}d_{\mathrm{ref}}^{\alpha }}+\sum_{i=1}^{N_{\mathrm{cell}}}\frac{p_{i}\beta_{i}}{d_{i}^{\alpha }}\right)\cdot \left(2^{\frac{R}{r_{s}B}}-1\right)\right)\\ & & \;\times \left[\sum_{n=1}^{N_{\mathrm{tx}}N_{\mathrm{rx}}}\frac{1}{(n-1)!}{\left\{\frac{r_{s}N_{\mathrm{tx}}d_{s}^{\alpha }}{p_{s}\beta_{s}}\left(\frac{N_{0}B}{k_{0}d_{\mathrm{ref}}^{\alpha }}+\sum_{i=1}^{N_{\mathrm{cell}}}\frac{p_{i}\beta_{i}}{d_{i}^{\alpha }}\right)\cdot \left(2^{\frac{R}{r_{s}B}}-1\right)\right\}}^{n-1}\right], \end{array}$$

(8) $$\begin{array}{ccc} & & P_{\mathrm{ostbc},\;p}\left(p_{s}\right)=1-\exp\left(-\frac{r_{s}N_{\mathrm{tx}}d_{s}^{\alpha }}{p_{s}\beta_{s}}\left(\frac{N_{0}B}{mk_{0}d_{\mathrm{ref}}^{\alpha }}+\sum_{i=1}^{N_{\mathrm{cell}}}\frac{p_{i}\beta_{i}}{d_{i}^{\alpha }}\;I_{m}\left(i\right)\right)\cdot \left(2^{\frac{mR}{r_{s}B}}-1\right)\right)\\ & & \;\;\times \left[\sum_{n=1}^{N_{\mathrm{tx}}N_{\mathrm{rx}}}\frac{1}{(n-1)!}{\left\{\frac{r_{s}N_{\mathrm{tx}}d_{s}^{\alpha }}{p_{s}\beta_{s}}\left(\frac{N_{0}B}{mk_{0}d_{\mathrm{ref}}^{\alpha }}+\sum_{i=1}^{N_{\mathrm{cell}}}\frac{p_{i}\beta_{i}}{d_{i}^{\alpha }}\;I_{m}\left(i\right)\right)\cdot \left(2^{\frac{mR}{r_{s}B}}-1\right)\right\}}^{n-1}\right], \end{array}$$

where we used the fact that $I_{1}\left(i\right)=1$$\left(i=1,\ldots ,N_{\mathrm{cell}}\right)$ for full FR. With this setup, as follows, we investigate the optimal full FR coverage in terms of the base station power.

**Theorem 1.**  *Consider the MISO/MIMO systems of OSTBC ($N_{\mathrm{tx}}\geq 2$, $N_{\mathrm{rx}}\geq 1$). For base station power $p_{s}>0$, the outage probabilities of full FR and partial FR satisfy the following:*

(9) $$\begin{array}{c} P_{\mathrm{ostbc},\;p}\left(p_{s}\right)<P_{\mathrm{ostbc},\;f}\left(p_{s}\right)\;\;\;\mathrm{for}\;\;R<R_{\mathrm{ostbc}}^{*},\;\;\;\;\\ P_{\mathrm{ostbc},\;p}\left(p_{s}\right)=P_{\mathrm{ostbc},\;f}\left(p_{s}\right)\;\;\;\mathrm{for}\;\;R=R_{\mathrm{ostbc}}^{*},\;\;\;\;\\ P_{\mathrm{ostbc},\;p}\left(p_{s}\right)>P_{\mathrm{ostbc},\;f}\left(p_{s}\right)\;\;\;\mathrm{for}\;\;R>R_{\mathrm{ostbc}}^{*},\;\;\;\;\; \end{array}$$

*where $P_{\mathrm{ostbc},\;p}\left(p_{s}\right)$ and $P_{\mathrm{ostbc},\;f}\left(p_{s}\right)$ are given by (8), and $R_{\mathrm{ostbc}}^{*}$ is the data rate satisfying the equality given by*

(10) $$\begin{array}{c} \sum_{k=1}^{m}2^{\frac{\left(m-k\right)R_{\mathrm{ostbc}}^{*}}{r_{s}B}}=\frac{\frac{N_{0}B}{k_{0}d_{\mathrm{ref}}^{\alpha }}+\sum_{i=1}^{N_{\mathrm{cell}}}\frac{p_{i}\beta_{i}}{d_{i}^{\alpha }}}{\;\frac{N_{0}B}{mk_{0}d_{\mathrm{ref}}^{\alpha }}+\sum_{i=1}^{N_{\mathrm{cell}}}\frac{p_{i}\beta_{i}}{d_{i}^{\alpha }}\;I_{m}\left(i\right)\;}\;. \end{array}$$

**Proof.**  We define the function f(x) as

(11) $$f\left(x\right)=1-e^{-x}\left(\sum_{n=1}^{N_{\mathrm{tx}}N_{\mathrm{rx}}}\frac{1}{(n-1)!}x^{n-1}\right).$$

It can be shown that $df\left(x\right)/dx=e^{-x}x^{N_{\mathrm{tx}}N_{\mathrm{rx}}-1}/(N_{\mathrm{tx}}N_{\mathrm{rx}}-1)!>0$ for x>0. Using (11), $P_{\mathrm{ostbc},\;f}\left(p_{s}\right)$ and $P_{\mathrm{ostbc},\;p}\left(p_{s}\right)$, given by (8), respectively, can be rewritten as

(12) $$P_{\mathrm{ostbc},\;f}\left(p_{s}\right)=f\left(u_{f}\left(p_{s}\right)\right)\;\;\mathrm{and}\;\;P_{\mathrm{ostbc},\;p}\left(p_{s}\right)=f\left(u_{p}\left(p_{s}\right)\right),$$

where $u_{f}\left(p_{s}\right)$ and $u_{p}\left(p_{s}\right)$ are given by

(13) $$\begin{array}{ccc} & & u_{f}\left(p_{s}\right)=\frac{r_{s}N_{\mathrm{tx}}d_{s}^{\alpha }}{p_{s}\beta_{s}}\left(\frac{N_{0}B}{k_{0}\;d_{\mathrm{ref}}^{\alpha }}+\sum_{i=1}^{N_{\mathrm{cell}}}\frac{p_{i}\beta_{i}}{d_{i}^{\alpha }}\right)\cdot \left(2^{\frac{R}{r_{s}B}}-1\right)>0,\\ & & u_{p}\left(p_{s}\right)=\frac{r_{s}N_{\mathrm{tx}}d_{s}^{\alpha }}{p_{s}\beta_{s}}\left(\frac{N_{0}B}{mk_{0}\;d_{\mathrm{ref}}^{\alpha }}+\sum_{i=1}^{N_{\mathrm{cell}}}\frac{p_{i}\beta_{i}}{d_{i}^{\alpha }}\;I_{m}\left(i\right)\right)\cdot \left(2^{\frac{mR}{r_{s}B}}-1\right)>0. \end{array}$$

From (13), $u_{f}\left(p_{s}\right)/u_{p}\left(p_{s}\right)$ can be expressed as

(14) $$\begin{array}{c} \frac{u_{f}\left(p_{s}\right)}{u_{p}\left(p_{s}\right)}=\frac{\frac{N_{0}B}{k_{0}d_{\mathrm{ref}}^{\alpha }}+\sum_{i=1}^{N_{\mathrm{cell}}}\frac{p_{i}\beta_{i}}{d_{i}^{\alpha }}}{\frac{N_{0}B}{mk_{0}d_{\mathrm{ref}}^{\alpha }}+\sum_{i=1}^{N_{\mathrm{cell}}}\frac{p_{i}\beta_{i}}{d_{i}^{\alpha }}\;I_{m}\left(i\right)}\cdot \frac{1}{\;\sum_{k=1}^{m}2^{\frac{(m-k)R}{r_{s}B}}\;}=\frac{\;\sum_{k=1}^{m}2^{\frac{\left(m-k\right)R_{\mathrm{ostbc}}^{*}}{r_{s}B}}\;}{\sum_{k=1}^{m}2^{\frac{(m-k)R}{r_{s}B}}}\;, \end{array}$$

where the second equality follows from the definition of $R_{\mathrm{ostbc}}^{*}$ given by (10). Since $2^{\frac{(m-k)R}{r_{s}B}}\;\left(k=1,\ldots ,m-1\right)$ is a strictly increasing function of *R*, and since df(x)/dx>0 for x>0, it follows from (12) that (9) holds. □

**Figure 3:**
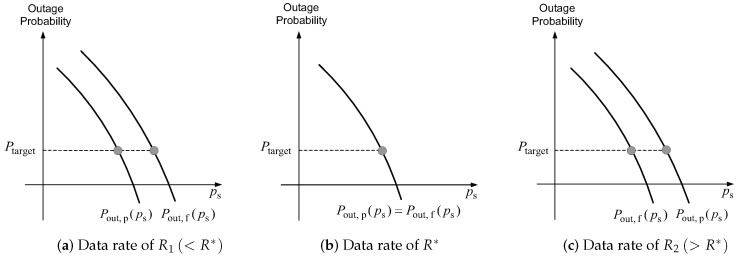
Outage probabilities of full and partial FRs for a given data rate. In this figure, $\left\{P_{\mathrm{out},\;f}\left(p_{s}\right),P_{\mathrm{out},\;p}\left(p_{s}\right),R^{*}\right\}$ respectively indicate $\left\{P_{\mathrm{otsbc},\;f}\left(p_{s}\right),P_{\mathrm{ostbc},\;p}\left(p_{s}\right),R_{\mathrm{ostbc}}^{*}\right\}$ for OSTBC, and $\left\{P_{\mathrm{vblast},\;f}\left(p_{s}\right),P_{\mathrm{vblast},\;p}\left(p_{s}\right),R_{\mathrm{vblast}}^{*}\right\}$ for V-BLAST.

Based on (9), for the data rates of $R_{1}<R_{\mathrm{ostbc}}^{*}<R_{2}$, the outage probabilities of full FR and partial FR are qualitatively depicted in Figure 3. In contrast to [22] (Figure 1), no error floors are shown for the outage probability curves since SINR, given by (5), approaches infinity as $p_{s}$ approaches infinity. Thus, a given target outage probability, denoted by $P_{\mathrm{target}}$, is achieved for any data rate, provided $p_{s}$ is sufficiently large. Figure 3 shows that the preference between full and partial FRs depends on the data rate. For a low data rate $R_{1}\;\left(<R_{\mathrm{ostbc}}^{*}\right)$, partial FR is preferable to full FR. For a high data rate $R_{2}\;\left(>R_{\mathrm{ostbc}}^{*}\right)$, however, full FR is preferable to partial FR. For a data rate of $R_{\mathrm{ostbc}}^{*}$, full and partial FRs are equally preferable. Furthermore, as shown in Figure 3, as the data rate increases, the preference shifts only from partial FR to full FR. Conversely, when the data rate decreases, the preference transitions only from full FR to partial FR.

**Figure 4:**
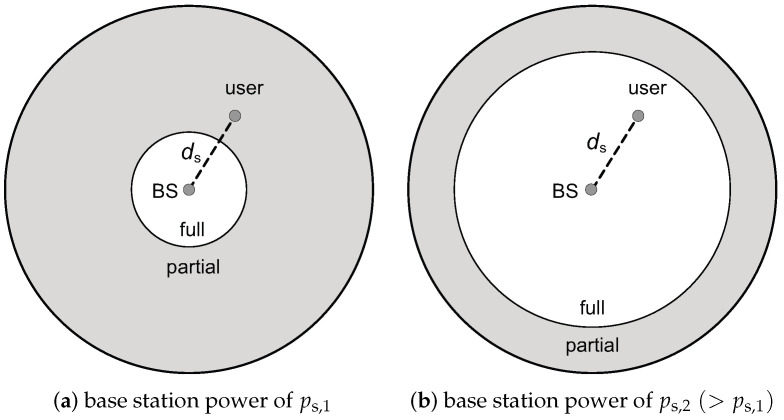
Change in the optimal full FR coverage with base station (BS) power adjustment. The gray area represents the partial FR region, while the white area indicates the full FR region. (**a**) Initially, the user’s location is within the partial FR region at a distance $d_{s}$ from the serving cell’s base station. (**b**) Following a power scaling, where the base station power increases $p_{s,2}/p_{s,1}$ (greater than 1) times in the serving cell, the user’s location transitions from the partial FR region to the full FR region.

Figure 4 depicts the change of the optimal full FR coverage in a single cell as the base station power in that cell is scaled. In Figure 4a, the base station power is $p_{s}=p_{s,1}$, and the user, which is at a distance $d_{s}$ from base station, is assumed to be in the partial FR region. As base station power increases to $p_{s}=p_{s,2}\;\left(>p_{s,1}\right)$, SINR, given by (5), also increases. As a result, a higher data rate can be employed, while a given target outage probability remains satisfied. As shown in Figure 3, the change in preference can only occur from partial FR to full FR as the data rate increases. As the point of this change, the user’s location, which is at a distance of $d_{s}$ from the base station, shifts from the partial FR to the full FR region. As a result, as illustrated in Figure 4b, the optimal full FR coverage extends.

Note that the change in the optimal full FR coverage does not necessarily follow the change in base station power. The optimal coverage changes if and only if the data rate increases from $R<R_{\mathrm{ostbc}}^{*}$ to $R>R_{\mathrm{ostbc}}^{*}$, or vice versa. Otherwise, the optimal full FR coverage stays the same even when the data rate changes (i.e., even when base station power changes). It therefore follows that the optimal full FR coverage, denoted by $d_{\mathrm{coverage}}^{*}$, is a non-decreasing function of base station power $p_{s}$. That is, we obtain

(15) $$\begin{array}{c} \frac{\partial d_{\mathrm{coverage}}^{*}}{\partial p_{s}}\geq 0. \end{array}$$

Equation (15) applies only to the serving cell, and it is assumed that the base station power in the neighboring cells is not scaled. Interestingly, the analytical results in this paper, given by Figure 4 and Equation (15), are the opposite to those in [22] (refer to Figure 2 and Equation (21)) that were derived for the case where the power of all base stations is scaled at the same rate.

We note that the results in this section hold for any partial FR factor M(=m>1), any signal bandwidth *B*, any path-loss exponent α, and any shadowing attenuation $\beta_{s}$ and $\beta_{i}$. In addition, a high signal-to-noise ratio (SNR) approximate expression of outage probability is not used in our analysis; the results are derived using the exact outage probabilities of $P_{\mathrm{ostbc},\;f}\left(p_{s}\right)$ and $P_{\mathrm{ostbc},\;p}\left(p_{s}\right)$ given by (8). Hence, the results hold for all SINRs. Lastly, we note that the results qualitatively shown in Figure 3 and Figure 4 apply to cellular networks of any topology with the arbitrary deployment of base stations. The cell shown in Figure 4 is a circular shape, but the results presented here apply to cells of any shape.

### 3.2. MIMO Systems of V-BLAST with a Zero-Forcing Linear Receiver

We next consider the case where the MIMO systems of V-BLAST with a zero-forcing linear receiver ($N_{\mathrm{rx}}\geq N_{\mathrm{tx}}\geq 2$) are adopted for the downlink transmission in the serving cell. In the V-BLAST approach, we make the assumption of pure spatial multiplexing as described in [31,32]. This assumption involves dividing the data into multiple substreams, with each substream undergoing independent temporal coding, thus simplifying the decoding process at the receiver. In this scheme, an outage event is defined when any of the substreams experiences an outage (that is, any of the subchannels cannot sustain the assigned data rate) [31]. Based on (1), the post-processing SINR at the user for the *n*th transmit stream ($n=1,\cdots ,N_{\mathrm{tx}}$) can be formulated as [28]

(16) $$\mathrm{SINR}=\frac{\;\;\frac{1}{N_{\mathrm{tx}}}p_{s}k_{0}{\left(\frac{d_{\mathrm{ref}}}{d_{s}}\right)}^{\alpha }\beta_{s}\lambda_{n}}{\frac{N_{0}B}{M}+\sum_{i=1}^{N_{\mathrm{cell}}}p_{i}k_{0}{\left(\frac{d_{\mathrm{ref}}}{d_{i}}\right)}^{\alpha }\beta_{i}I_{M}\left(i\right)}\;,$$

where $\lambda_{n}$ is a chi-square random variable with $2(N_{\mathrm{rx}}-N_{\mathrm{tx}}+1)$ degrees of freedom. Thus, it can be shown that the outage probability for the V-BLAST is expressed as [33]

(17) $$P_{\mathrm{vblast}}\left(p_{s}\right)=\mathrm{Pr}\left[\overset{N_{\mathrm{tx}}}{\underset{n=1}{⋃}}\left\{\frac{B}{M}\log_{2}\left(1+\frac{\;\;\frac{1}{N_{\mathrm{tx}}}p_{s}k_{0}{\left(\frac{d_{\mathrm{ref}}}{d_{s}}\right)}^{\alpha }\beta_{s}\lambda_{n}}{\frac{N_{0}B}{M}+\sum_{i=1}^{N_{\mathrm{cell}}}p_{i}k_{0}{\left(\frac{d_{\mathrm{ref}}}{d_{i}}\right)}^{\alpha }\beta_{i}I_{M}\left(i\right)}\right)<\frac{R}{N_{\mathrm{tx}}}\right\}\right],$$

where $R/N_{\mathrm{tx}}$ is the data rate (bits/s) assigned to each of the $N_{\mathrm{tx}}$ transmit streams and the capacity formula is used for each stream. Equation (17) can be rewritten as [33]

(18) $$\begin{array}{c} \;P_{\mathrm{vblast}}\left(p_{s}\right)=1-\mathrm{Pr}\left[\overset{N_{\mathrm{tx}}}{\underset{n=1}{⋂}}\left\{\lambda_{n}\geq \frac{N_{\mathrm{tx}}d_{s}^{\alpha }}{p_{s}\beta_{s}}\left(\frac{N_{0}B}{Mk_{0}d_{\mathrm{ref}}^{\alpha }}+\sum_{i=1}^{N_{\mathrm{cell}}}\frac{p_{i}\beta_{i}}{d_{i}^{\alpha }}I_{M}\left(i\right)\right)\cdot \left(2^{\frac{MR}{N_{\mathrm{tx}}B}}-1\right)\right\}\right]. \end{array}$$

The CDF of $\lambda_{n}$ is given by $F_{\lambda_{n}}\left(x\right)=1-e^{-x}\left(\sum_{n=1}^{N_{\mathrm{rx}}-N_{\mathrm{tx}}+1}x^{n-1}/\left(n-1\right)!\right)$. Based on the assumption that $\lambda_{n}$s are independent for a zero-forcing linear receiver [34,35,36], it can be shown that the outage probabilities for the full and partial FRs are given by [37]

$$\begin{array}{ccc} & & \;P_{\mathrm{vblast},\;f}\left(p_{s}\right)=1-[\exp\left(-\frac{N_{\mathrm{tx}}d_{s}^{\alpha }}{p_{s}\beta_{s}}\left(\frac{N_{0}B}{k_{0}d_{\mathrm{ref}}^{\alpha }}+\sum_{i=1}^{N_{\mathrm{cell}}}\frac{p_{i}\beta_{i}}{d_{i}^{\alpha }}\right)\cdot \left(2^{\frac{R}{N_{\mathrm{tx}}B}}-1\right)\right)\\ & & \;\times \left\{\sum_{n=1}^{N_{\mathrm{rx}}-N_{\mathrm{tx}}+1}\frac{1}{(n-1)!}{\left(\frac{N_{\mathrm{tx}}d_{s}^{\alpha }}{p_{s}\beta_{s}}\left(\frac{N_{0}B}{k_{0}d_{\mathrm{ref}}^{\alpha }}+\sum_{i=1}^{N_{\mathrm{cell}}}\frac{p_{i}\beta_{i}}{d_{i}^{\alpha }}\right)\cdot \left(2^{\frac{R}{N_{\mathrm{tx}}B}}-1\right)\right)}^{n-1}\right\}{]}^{N_{\mathrm{tx}}}, \end{array}$$

(19) $$\begin{array}{ccc} & & \;P_{\mathrm{vblast},\;p}\left(p_{s}\right)=1-[\exp\left(-\frac{N_{\mathrm{tx}}d_{s}^{\alpha }}{p_{s}\beta_{s}}\left(\frac{N_{0}B}{mk_{0}d_{\mathrm{ref}}^{\alpha }}+\sum_{i=1}^{N_{\mathrm{cell}}}\frac{p_{i}\beta_{i}}{d_{i}^{\alpha }}I_{m}\left(i\right)\right)\cdot \left(2^{\frac{mR}{N_{\mathrm{tx}}B}}-1\right)\right)\\ & & \;\times \left\{\sum_{n=1}^{N_{\mathrm{rx}}-N_{\mathrm{tx}}+1}\frac{1}{(n-1)!}{\left(\frac{N_{\mathrm{tx}}d_{s}^{\alpha }}{p_{s}\beta_{s}}\left(\frac{N_{0}B}{mk_{0}d_{\mathrm{ref}}^{\alpha }}+\sum_{i=1}^{N_{\mathrm{cell}}}\frac{p_{i}\beta_{i}}{d_{i}^{\alpha }}I_{m}\left(i\right)\right)\cdot \left(2^{\frac{mR}{N_{\mathrm{tx}}B}}-1\right)\right)}^{n-1}\right\}{]}^{N_{\mathrm{tx}}}.\;\;\;\;\; \end{array}$$

**Theorem 2.**  *Consider the MIMO systems of V-BLAST with a zero-forcing linear receiver ($N_{\mathrm{rx}}\geq N_{\mathrm{tx}}\geq 2$). For base station power $p_{s}>0$, the outage probabilities of full and partial FRs satisfy*

(20) $$\begin{array}{c} P_{\mathrm{vblast},\;p}\left(p_{s}\right)<P_{\mathrm{vblast},\;f}\left(p_{s}\right)\;\;\;\mathrm{for}\;\;R<R_{\mathrm{vblast}}^{*},\;\;\;\;\\ P_{\mathrm{vblast},\;p}\left(p_{s}\right)=P_{\mathrm{vblast},\;f}\left(p_{s}\right)\;\;\;\mathrm{for}\;\;R=R_{\mathrm{vblast}}^{*},\;\;\;\;\\ P_{\mathrm{vblast},\;p}\left(p_{s}\right)>P_{\mathrm{vblast},\;f}\left(p_{s}\right)\;\;\;\mathrm{for}\;\;R>R_{\mathrm{vblast}}^{*},\;\;\;\;\; \end{array}$$

*where $P_{\mathrm{vblast},\;p}\left(p_{s}\right)$ and $P_{\mathrm{vblast},\;f}\left(p_{s}\right)$ are given by (19), and $R_{\mathrm{vblast}}^{*}$ is the data rate that satisfies the following equality:*

(21) $$\begin{array}{c} \sum_{k=1}^{m}2^{\frac{\left(m-k\right)R_{\mathrm{vblast}}^{*}}{N_{\mathrm{tx}}B}}=\frac{\frac{N_{0}B}{k_{0}d_{\mathrm{ref}}^{\alpha }}+\sum_{i=1}^{N_{\mathrm{cell}}}\frac{p_{i}\beta_{i}}{d_{i}^{\alpha }}}{\;\frac{N_{0}B}{mk_{0}d_{\mathrm{ref}}^{\alpha }}+\sum_{i=1}^{N_{\mathrm{cell}}}\frac{p_{i}\beta_{i}}{d_{i}^{\alpha }}\;I_{m}\left(i\right)\;}\;. \end{array}$$

**Proof.**  We define the function g(x) as

(22) $$g\left(x\right)=1-{\left[e^{-x}\left(\sum_{n=1}^{N_{\mathrm{rx}}-N_{\mathrm{tx}}+1}\frac{1}{(n-1)!}x^{n-1}\right)\right]}^{N_{\mathrm{tx}}}.$$

It can be shown for x>0 that $dg\left(x\right)/dx=N_{\mathrm{tx}}\;x^{N_{\mathrm{rx}}-N_{\mathrm{tx}}}e^{-N_{\mathrm{tx}}x}$${\left(\sum_{n=1}^{N_{\mathrm{rx}}-N_{\mathrm{tx}}+1}x^{n-1}/\left(n-1\right)!\right)}^{N_{\mathrm{tx}}-1}/(N_{\mathrm{rx}}-N_{\mathrm{tx}})!>0$. Using g(x), $P_{\mathrm{vblast},\;f}\left(p_{s}\right)$ and $P_{\mathrm{vblast},\;p}\left(p_{s}\right)$, given by (19), can be expressed as

(23) $$P_{\mathrm{vblast},\;f}\left(p_{s}\right)=g\left(v_{f}\left(p_{s}\right)\right)\;\;\mathrm{and}\;\;P_{\mathrm{vblast},\;p}\left(p_{s}\right)=g\left(v_{p}\left(p_{s}\right)\right),$$

where $v_{f}\left(p_{s}\right)$ and $v_{p}\left(p_{s}\right)$ are given by

(24) $$\begin{array}{ccc} & & v_{f}\left(p_{s}\right)=\frac{N_{\mathrm{tx}}d_{s}^{\alpha }}{p_{s}\beta_{s}}\left(\frac{N_{0}B}{k_{0}d_{\mathrm{ref}}^{\alpha }}+\sum_{i=1}^{N_{\mathrm{cell}}}\frac{p_{i}\beta_{i}}{d_{i}^{\alpha }}\right)\cdot \left(2^{\frac{R}{N_{\mathrm{tx}}B}}-1\right)>0,\\ & & v_{p}\left(p_{s}\right)=\frac{N_{\mathrm{tx}}d_{s}^{\alpha }}{p_{s}\beta_{s}}\left(\frac{N_{0}B}{mk_{0}d_{\mathrm{ref}}^{\alpha }}+\sum_{i=1}^{N_{\mathrm{cell}}}\frac{p_{i}\beta_{i}}{d_{i}^{\alpha }}\;I_{m}\left(i\right)\right)\cdot \left(2^{\frac{mR}{N_{\mathrm{tx}}B}}-1\right)>0. \end{array}$$

From (24), $v_{f}\left(p_{s}\right)/v_{p}\left(p_{s}\right)$ can be expressed as

(25) $$\begin{array}{c} \frac{v_{f}\left(p_{s}\right)}{v_{p}\left(p_{s}\right)}=\frac{\frac{N_{0}B}{k_{0}d_{\mathrm{ref}}^{\alpha }}+\sum_{i=1}^{N_{\mathrm{cell}}}\frac{p_{i}\beta_{i}}{d_{i}^{\alpha }}}{\frac{N_{0}B}{mk_{0}d_{\mathrm{ref}}^{\alpha }}+\sum_{i=1}^{N_{\mathrm{cell}}}\frac{p_{i}\beta_{i}}{d_{i}^{\alpha }}\;I_{m}\left(i\right)}\cdot \frac{1}{\;\sum_{k=1}^{m}2^{\frac{(m-k)R}{N_{\mathrm{tx}}B}}\;}=\frac{\;\sum_{k=1}^{m}2^{\frac{\left(m-k\right)R_{\mathrm{vblast}}^{*}}{N_{\mathrm{tx}}B}}\;}{\sum_{k=1}^{m}2^{\frac{(m-k)R}{N_{\mathrm{tx}}B}}}\;, \end{array}$$

where the second equality follows from the definition of $R_{\mathrm{vblast}}^{*}$ given by (21). Since $2^{\frac{(m-k)R}{N_{\mathrm{tx}}B}}\;\left(k=1,\ldots ,m-1\right)$ is a strictly increasing function of *R*, and since dg(x)/dx>0 for x>0, it follows from (23) that (20) holds. □

Theorem 2 indicates that the results for OSTBC, which are shown in Figure 3 and Figure 4, and Equation (15), also hold for V-BLAST with a zero-forcing linear receiver.

### 3.3. SISO Systems

If we set the number of transmit antennas and the spatial multiplexing rate to $N_{\mathrm{tx}}=1$ and $r_{s}=1$, respectively, all the results in Section 3.1 can be shown to hold for SISO systems.

## 4. Numerical Evaluation

### 4.1. MISO or MIMO Systems of OSTBC

To begin, we rewrite the outage probabilities for OSTBC, given by (8), as a function of SINR instead of base station power $p_{s}$. From (5), the average SINR of full FR for OSTBC can be expressed as $\gamma_{\mathrm{ostbc}}=E\left[\frac{1}{r_{s}N_{\mathrm{tx}}}p_{s}k_{0}{\left(\frac{d_{\mathrm{ref}}}{d_{s}}\right)}^{\alpha }\beta_{s}{∥H∥}_{F}^{2}/\left(N_{0}B+\sum_{i=1}^{N_{\mathrm{cell}}}p_{i}k_{0}{\left(\frac{d_{\mathrm{ref}}}{d_{i}}\right)}^{\alpha }\beta_{i}\right)\right]$ $=p_{s}k_{0}d_{\mathrm{ref}}^{\alpha }\beta_{s}N_{\mathrm{rx}}/$$r_{s}d_{s}^{\alpha }\left(N_{0}B+\sum_{i=1}^{N_{\mathrm{cell}}}p_{i}k_{0}{\left(\frac{d_{\mathrm{ref}}}{d_{i}}\right)}^{\alpha }\beta_{i}\right)$, where the second equality follows from the fact that the entries of the $N_{\mathrm{rx}}\times N_{\mathrm{tx}}$ channel matrix H are distributed as CN(0,1). Then, the outage probabilities, given by (8), can be rewritten as a function of $\gamma_{\mathrm{ostbc}}$ as

$$\begin{array}{ccc} & & \;P_{\mathrm{ostbc},\;f}\left(\gamma_{\mathrm{ostbc}}\right)=\\ & & \;1-\exp\left(-\frac{N_{\mathrm{tx}}N_{\mathrm{rx}}}{\gamma_{\mathrm{ostbc}}\left(N_{0}B+\sum_{i=1}^{N_{\mathrm{cell}}}p_{i}k_{0}{\left(\frac{d_{\mathrm{ref}}}{d_{i}}\right)}^{\alpha }\beta_{i}\right)}\left(N_{0}B+k_{0}d_{\mathrm{ref}}^{\alpha }\sum_{i=1}^{N_{\mathrm{cell}}}\frac{p_{i}\beta_{i}}{d_{i}^{\alpha }}\right)\cdot \left(2^{\frac{R}{r_{s}B}}-1\right)\right)\\ & & \;\times [\sum_{n=1}^{N_{\mathrm{tx}}N_{\mathrm{rx}}}\frac{1}{(n-1)!}\{\frac{N_{\mathrm{tx}}N_{\mathrm{rx}}}{\gamma_{\mathrm{ostbc}}\left(N_{0}B+\sum_{i=1}^{N_{\mathrm{cell}}}p_{i}k_{0}{\left(\frac{d_{\mathrm{ref}}}{d_{i}}\right)}^{\alpha }\beta_{i}\right)}\left(N_{0}B+k_{0}d_{\mathrm{ref}}^{\alpha }\sum_{i=1}^{N_{\mathrm{cell}}}\frac{p_{i}\beta_{i}}{d_{i}^{\alpha }}\right)\\ & & \;\times \left(2^{\frac{R}{r_{s}B}}-1\right){\}}^{n-1}], \end{array}$$

(26) $$\begin{array}{ccc} & & \;P_{\mathrm{ostbc},\;p}\left(\gamma_{\mathrm{ostbc}}\right)=\\ & & \;1-\exp\left(-\frac{N_{\mathrm{tx}}N_{\mathrm{rx}}}{\gamma_{\mathrm{ostbc}}\left(N_{0}B+\sum_{i=1}^{N_{\mathrm{cell}}}p_{i}k_{0}{\left(\frac{d_{\mathrm{ref}}}{d_{i}}\right)}^{\alpha }\beta_{i}\right)}\left(\frac{N_{0}B}{m}+k_{0}d_{\mathrm{ref}}^{\alpha }\sum_{i=1}^{N_{\mathrm{cell}}}\frac{p_{i}\beta_{i}}{d_{i}^{\alpha }}\;I_{m}\left(i\right)\right)\cdot \left(2^{\frac{mR}{r_{s}B}}-1\right)\right)\\ & & \;\times [\sum_{n=1}^{N_{\mathrm{tx}}N_{\mathrm{rx}}}\frac{1}{(n-1)!}\{\frac{N_{\mathrm{tx}}N_{\mathrm{rx}}}{\gamma_{\mathrm{ostbc}}\left(N_{0}B+\sum_{i=1}^{N_{\mathrm{cell}}}p_{i}k_{0}{\left(\frac{d_{\mathrm{ref}}}{d_{i}}\right)}^{\alpha }\beta_{i}\right)}\left(\frac{N_{0}B}{m}+k_{0}d_{\mathrm{ref}}^{\alpha }\sum_{i=1}^{N_{\mathrm{cell}}}\frac{p_{i}\beta_{i}}{d_{i}^{\alpha }}\;I_{m}\left(i\right)\right)\\ & & \;\times \left(2^{\frac{mR}{r_{s}B}}-1\right){\}}^{n-1}]. \end{array}$$

**Figure 5:**
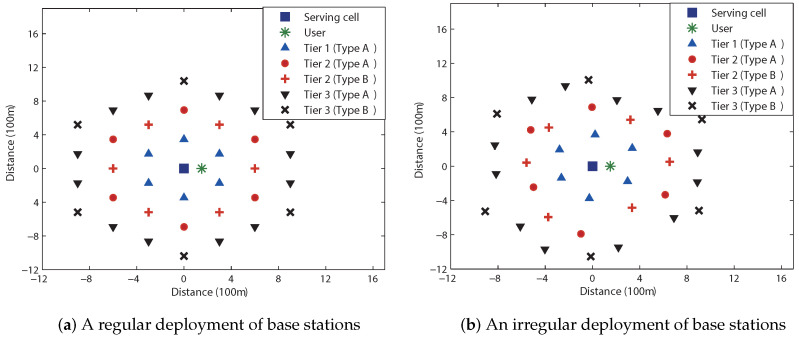
Hexagonal cellular networks comprising 37 cells with the partial FR factor of M=3. Cells are categorized into two types: Type A cells generate interference exclusively for users within the full FR region. Type B cells introduce interference for users in both the full FR and the partial FR regions.

**Figure 6:**
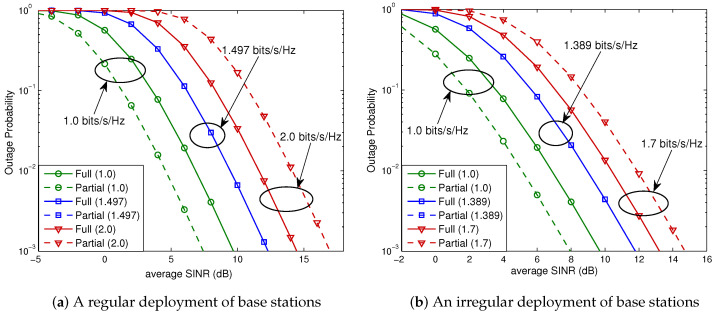
Outage probabilities for a 2×2 OSTBC scheme in a hexagonal cellular system. Solid curves denote the outage probabilities for full FR, while the dashed curves denote those for partial FR. (**a**) Equation (27) yields $R_{\mathrm{ostbc}}^{*}/B=1.497$ (bits/s/Hz). (**b**) From (27), we obtain $R_{\mathrm{ostbc}}^{*}/B=1.389$ (bits/s/Hz).

In this evaluation, as an example, system parameters are set to $d_{\mathrm{ref}}=5$ (m), α=4, B=10 (MHz), $N_{0}=-174$ (dBm/Hz), $p_{1}=\cdots =p_{N_{\mathrm{cell}}}=1$ (watt), $\beta_{s}=\beta_{1}=\cdots =\beta_{N_{\mathrm{cell}}}=1$, and $k_{0}={\left(3\cdot 10^{8}/4\pi \;d_{\mathrm{ref}}\;f_{c}\right)}^{2}$, where $f_{c}=5$ (GHz) is the carrier frequency. We first consider the hexagonal cellular network that comprises 37 base stations (i.e., $N_{\mathrm{cell}}=36$), which are deployed either in a regular or in an irregular fashion. The base station layouts used for this evaluation are shown in Figure 5, where the partial FR factor is M=3 and we can divide 36 neighboring cells into three tiers according to their distances from the serving cell. Cells that only interfere with the user located in the full FR region of the serving cell are referred to as *Type A*. *Type B* cells are those that interfere with users in both the full FR and partial FR regions. The numerical results for the regular base station layout are depicted in Figure 6a; for the spectral efficiencies of R/B=1.0, 1.497, and 2.0 (bits/s/Hz) (or, equivalently, for the data rates of R=10.0, 14.97, and 20.0 (Mbits/s)), the outage probabilities for a 2×2 MIMO system of OSTBC, given by (26), are evaluated. Substituting m=3 (i.e., partial FR factor of 3) into (10), we obtain

(27) $$R_{\mathrm{ostbc}}^{*}=r_{s}B\left[\log_{2}\left(-1+\sqrt{4\cdot \frac{\frac{N_{0}B}{k_{0}d_{\mathrm{ref}}^{\alpha }}+\sum_{i=1}^{N_{\mathrm{cell}}}\frac{p_{i}\beta_{i}}{d_{i}^{\alpha }}}{\;\frac{N_{0}B}{3k_{0}d_{\mathrm{ref}}^{\alpha }}+\sum_{i=1}^{N_{\mathrm{cell}}}\frac{p_{i}\beta_{i}}{d_{i}^{\alpha }}\;I_{3}\left(i\right)\;}-3}\right)-1\right].$$

In this case, from (27), we obtain $R_{\mathrm{ostbc}}^{*}/B=1.497$ (bits/s/Hz) (i.e., $R_{\mathrm{ostbc}}^{*}=14.97$ (Mbits/s)). As shown in Figure 6a, if the spectral efficiency is lower than $R_{\mathrm{ostbc}}^{*}/B$, partial FR is preferable for all SNRs (i.e., for all base station powers), while full FR is preferable if the spectral efficiency is higher than $R_{\mathrm{ostbc}}^{*}/B$. On the other hand, for the spectral efficiency equal to $R_{\mathrm{ostbc}}^{*}/B$, the full and partial FR schemes perform exactly the same. Note that these observations were predicted by the analysis given by (9) of Theorem 1. We next consider a hexagonal cellular network with an irregular layout of base stations shown in Figure 5b. The associated outage probabilities for R/B=1.0, 1.389, and 1.7 (bits/s/Hz) are shown in Figure 6b. For this case, (27) yields $R_{\mathrm{ostbc}}^{*}/B=1.389$ (bits/s/Hz). It is seen that the analytical results given by (9) of Theorem 1 also hold for a hexagonal system with an irregular base station layout.

**Figure 7:**
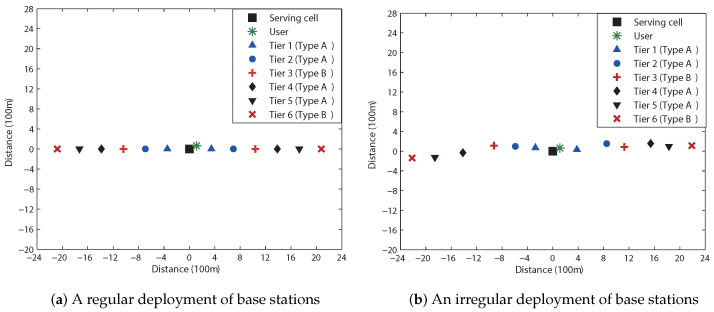
Linear cellular networks comprising 13 cells with the partial FR factor of M=3. Cells are categorized into two types: Type A cells yield interference exclusively for users within the full FR region. Type B cells produce interference for users in both the full FR and the partial FR regions. The serving cell, positioned at the center, is identified by a square symbol, and the user in close proximity to the serving cell is denoted by an asterisk symbol.

**Figure 8:**
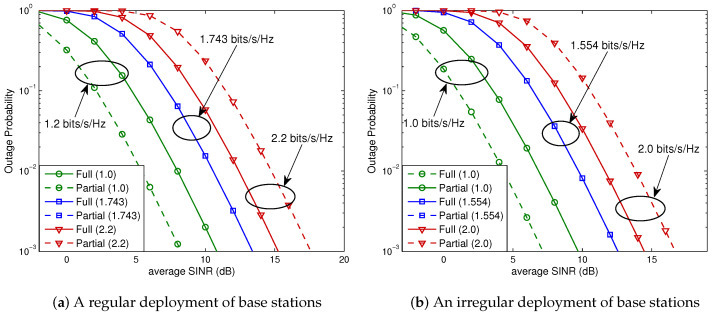
Outage probabilities for a 2×2 OSTBC scheme in a linear cellular system. Solid curves denote the outage probabilities for full FR, while the dashed curves denote those for partial FR. (**a**) We obtain $R_{\mathrm{ostbc}}^{*}/B=1.743$ (bits/s/Hz) from (27). (**b**) Equation (27) yields $R_{\mathrm{ostbc}}^{*}/B=1.554$ (bits/s/Hz).

We next assess the outage probabilities for a linear cellular system (e.g., a highway scenario). The system comprised of 13 cells is illustrated in Figure 7, with a partial FR factor set to M=3. The 12 neighboring cells can be categorized into 6 tiers based on their proximity to the serving cell. Cells that exclusively cause interference to users within the full FR region of the serving cell are labeled as *Type A*, and *Type B* cells interfere with users in both the full FR and partial FR regions. The corresponding outage probabilities are presented in Figure 8, showing that Theorem 1 is also valid for a linear cellular system, irrespective of whether the base stations are deployed in a regular pattern or not.

### 4.2. MIMO Systems of V-BLAST with a Zero-Forcing Linear Receiver

From (16), the average SINR of full FR for the *n*th transmit stream of V-BLAST ($k=1,\ldots ,N_{\mathrm{tx}}$) can be expressed as $\gamma_{\mathrm{vblast}}=E\left[\frac{1}{N_{\mathrm{tx}}}p_{s}k_{0}{\left(\frac{d_{\mathrm{ref}}}{d_{s}}\right)}^{\alpha }\beta_{s}\lambda_{n}/\left(N_{0}B+\sum_{i=1}^{N_{\mathrm{cell}}}p_{i}k_{0}{\left(\frac{d_{\mathrm{ref}}}{d_{i}}\right)}^{\alpha }\beta_{i}\right)\right]$ $=p_{s}k_{0}d_{\mathrm{ref}}^{\alpha }\beta_{s}\left(N_{\mathrm{rx}}-N_{\mathrm{tx}}+1\right)/$$N_{\mathrm{tx}}d_{s}^{\alpha }\left(N_{0}B+\sum_{i=1}^{N_{\mathrm{cell}}}p_{i}k_{0}{\left(\frac{d_{\mathrm{ref}}}{d_{i}}\right)}^{\alpha }\beta_{i}\right)$, where the second equality follows from the fact that $\lambda_{n}$ is a chi-square random variable with $2(N_{\mathrm{rx}}-N_{\mathrm{tx}}+1)$ degrees of freedom. The outage probabilities, given by (19), can be rewritten as a function of $\gamma_{\mathrm{vblast}}$ as

$$\begin{array}{ccc} & & \;P_{\mathrm{vblast},\;f}\left(\gamma_{\mathrm{vblast}}\right)=1-\\ & & \;[\exp\left(-\frac{N_{\mathrm{rx}}-N_{\mathrm{tx}}+1}{\gamma_{\mathrm{vblast}}\left(N_{0}B+\sum_{i=1}^{N_{\mathrm{cell}}}p_{i}k_{0}{\left(\frac{d_{\mathrm{ref}}}{d_{i}}\right)}^{\alpha }\beta_{i}\right)}\left(N_{0}B+k_{0}d_{\mathrm{ref}}^{\alpha }\sum_{i=1}^{N_{\mathrm{cell}}}\frac{p_{i}\beta_{i}}{d_{i}^{\alpha }}\right)\cdot \left(2^{\frac{R}{N_{\mathrm{tx}}B}}-1\right)\right)\\ & & \;\times \{\sum_{n=1}^{N_{\mathrm{rx}}-N_{\mathrm{tx}}+1}\frac{1}{(n-1)!}(\frac{N_{\mathrm{rx}}-N_{\mathrm{tx}}+1}{\gamma_{\mathrm{vblast}}\left(N_{0}B+\sum_{i=1}^{N_{\mathrm{cell}}}p_{i}k_{0}{\left(\frac{d_{\mathrm{ref}}}{d_{i}}\right)}^{\alpha }\beta_{i}\right)}\left(N_{0}B+k_{0}d_{\mathrm{ref}}^{\alpha }\sum_{i=1}^{N_{\mathrm{cell}}}\frac{p_{i}\beta_{i}}{d_{i}^{\alpha }}\right)\\ & & \;\times \left(2^{\frac{R}{N_{\mathrm{tx}}B}}-1\right){)}^{n-1}\}{]}^{N_{\mathrm{tx}}}, \end{array}$$

(28) $$\begin{array}{ccc} & & \;P_{\mathrm{vblast},\;p}\left(\gamma_{\mathrm{vblast}}\right)=1-\\ & & \;[\exp\left(-\frac{N_{\mathrm{rx}}-N_{\mathrm{tx}}+1}{\gamma_{\mathrm{vblast}}\left(N_{0}B+\sum_{i=1}^{N_{\mathrm{cell}}}p_{i}k_{0}{\left(\frac{d_{\mathrm{ref}}}{d_{i}}\right)}^{\alpha }\beta_{i}\right)}\left(\frac{N_{0}B}{m}+k_{0}d_{\mathrm{ref}}^{\alpha }\sum_{i=1}^{N_{\mathrm{cell}}}\frac{p_{i}\beta_{i}}{d_{i}^{\alpha }}I_{m}\left(i\right)\right)\cdot \left(2^{\frac{mR}{N_{\mathrm{tx}}B}}-1\right)\right)\\ & & \;\times \{\sum_{n=1}^{N_{\mathrm{rx}}-N_{\mathrm{tx}}+1}\frac{1}{(n-1)!}(\frac{N_{\mathrm{rx}}-N_{\mathrm{tx}}+1}{\gamma_{\mathrm{vblast}}\left(N_{0}B+\sum_{i=1}^{N_{\mathrm{cell}}}p_{i}k_{0}{\left(\frac{d_{\mathrm{ref}}}{d_{i}}\right)}^{\alpha }\beta_{i}\right)}\left(\frac{N_{0}B}{m}+k_{0}d_{\mathrm{ref}}^{\alpha }\sum_{i=1}^{N_{\mathrm{cell}}}\frac{p_{i}\beta_{i}}{d_{i}^{\alpha }}I_{m}\left(i\right)\right)\\ & & \;\times \left(2^{\frac{mR}{N_{\mathrm{tx}}B}}-1\right){)}^{n-1}\}{]}^{N_{\mathrm{tx}}}. \end{array}$$

**Figure 9:**
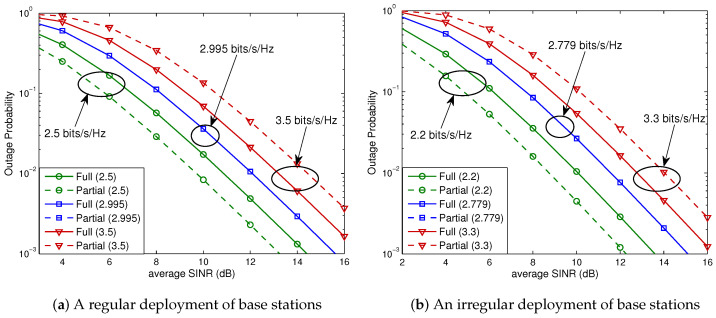
Outage probabilities for a 2×4 V-BLAST scheme in a hexagonal cellular system. Solid curves denote the outage probabilities for full FR, while the dashed curves denote those for partial FR. (**a**) $R_{\mathrm{vblast}}^{*}/B=2.995$ (bits/s/Hz). (**b**) $R_{\mathrm{vblast}}^{*}/B=2.779$ (bits/s/Hz).

**Figure 10:**
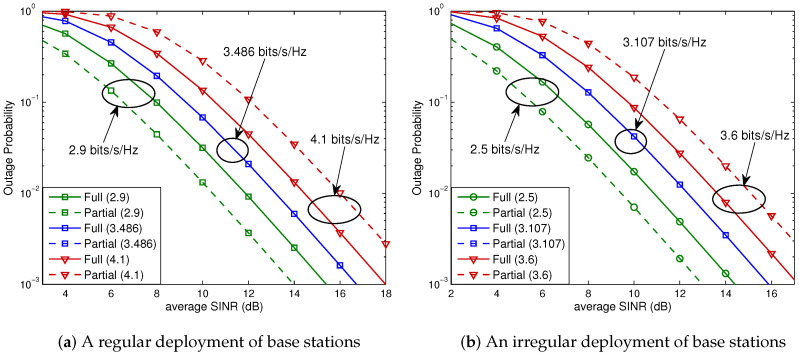
Outage probabilities for a 2×4 V-BLAST scheme in a linear cellular system. Solid curves denote the outage probabilities for full FR, while the dashed curves denote those for partial FR. (**a**) $R_{\mathrm{vblast}}^{*}=3.486$ (bits/s/Hz). (**b**) $R_{\mathrm{vblast}}^{*}=3.107$ (bits/s/Hz).

With the same setup as that used for OSTBC, we evaluate the outage probabilities of V-BLAST, given by (28), for a 2×4 MIMO system. The results for hexagonal and linear cellular networks are shown in Figure 9 and Figure 10, respectively. It is observed that the analytical results given by (20) of Theorem 2 also hold for V-BLAST with a zero-forcing linear receiver.

## 5. Conclusions

The full FR coverage, which distinguishes the full FR region from the partial FR region within a FFR cell, significantly affects the system performance. In this paper, we studied the optimal full FR coverage in MIMO FFR cellular networks. It is analytically shown that when the base station power in a single cell is scaled, the optimal full FR coverage in that cell is a non-decreasing function of power. Interestingly, these results are the exact opposite of the recent results studied in [22], which were obtained for the case where the power of all base stations is scaled at the same rate. Our result is purely analytical and provides us with a more profound understanding of the intricate relationship between critical FFR parameters of full FR coverage and power.

Note that our results are proven for any number of antennas in the MIMO systems of OSTBC and V-BLAST with a zero-forcing linear receiver, any partial FR factor, any frequency bandwidth, any path-loss exponent, and any shadowing attenuation. More importantly, in contrast to most previous studies, our results hold for arbitrary cellular topologies with any deployment of base stations. Further, our results are proven by the use of exact outage probability expressions instead of high SNR approximation. Future work may include the analysis of the optimal full FR coverage in soft frequency reuse (SFR) systems, in relation to the power control factor that is used to boost the SINR of users staying at the partial FR region.

## Data Availability

Not applicable.
